# Endothelial actions of atrial natriuretic peptide prevent pulmonary hypertension in mice

**DOI:** 10.1007/s00395-016-0541-x

**Published:** 2016-02-24

**Authors:** Franziska Werner, Baktybek Kojonazarov, Birgit Gaßner, Marco Abeßer, Kai Schuh, Katharina Völker, Hideo A. Baba, Bhola K. Dahal, Ralph T. Schermuly, Michaela Kuhn

**Affiliations:** 1 Physiologisches Institut der Universität Würzburg, Röntgenring 9, 97070 Würzburg, Germany; 2 Department of Internal Medicine, University of Gießen and Marburg Lung Center (UGMLC), Justus-Liebig University Gießen, Giessen, Germany; 3 German Center for Lung Research, Heidelberg, Germany; 4 Institute of Pathology, University Hospital of Essen, University of Duisburg-Essen, Essen, Germany

**Keywords:** Atrial natriuretic peptide, Endothelium, Guanylyl cyclase-A, Cyclic GMP, Pulmonary hypertension

## Abstract

The cardiac hormone atrial natriuretic peptide (ANP) regulates systemic and pulmonary arterial blood pressure by activation of its cyclic GMP-producing guanylyl cyclase-A (GC-A) receptor. In the lung, these hypotensive effects were mainly attributed to smooth muscle-mediated vasodilatation. It is unknown whether pulmonary endothelial cells participate in the homeostatic actions of ANP. Therefore, we analyzed GC-A/cGMP signalling in lung endothelial cells and the cause and functional impact of lung endothelial GC-A dysfunction. Western blot and cGMP determinations showed that cultured human and murine pulmonary endothelial cells exhibit prominent GC-A expression and activity which were markedly blunted by hypoxia, a condition known to trigger pulmonary hypertension (PH). To elucidate the consequences of impaired endothelial ANP signalling, we studied mice with genetic endothelial cell-restricted ablation of the GC-A receptor (EC GC-A KO). Notably, EC GC-A KO mice exhibit PH already under resting, normoxic conditions, with enhanced muscularization of small arteries and perivascular infiltration of inflammatory cells. These alterations were aggravated on exposure of mice to chronic hypoxia. Lung endothelial GC-A dysfunction was associated with enhanced expression of angiotensin converting enzyme (ACE) and increased pulmonary levels of Angiotensin II. Angiotensin II/AT_1_-blockade with losartan reversed pulmonary vascular remodelling and perivascular inflammation of EC GC-A KO mice, and prevented their increment by chronic hypoxia. This experimental study indicates that endothelial effects of ANP are critical to prevent pulmonary vascular remodelling and PH. *Chronic* endothelial ANP/GC-A dysfunction, e.g. provoked by hypoxia, is associated with activation of the ACE–angiotensin pathway in the lung and PH.

## Introduction

Pulmonary hypertension (PH) is a complex and multifactorial disease which leads to overload of the right ventricle (RV) and right heart failure. Pulmonary vasoconstriction, endothelial cell (EC) dysfunction, vascular thickening, inflammation and thrombosis contribute to disease progression in idiopathic and other forms of PH [3, 21].

The cardiac hormone atrial natriuretic peptide (ANP), via its cyclic GMP (cGMP)-synthesizing transmembrane guanylyl cyclase A (GC-A) receptor, has critical functions in the maintenance of systemic arterial blood pressure [6, 42] and also regulates pulmonary arterial blood pressure. Hence, global inactivation of the genes encoding ANP or GC-A increased resting pulmonary arterial pressure in mice [28, 29] or the susceptibility to hypoxia-induced PH [58]. Conversely, infusion of synthetic ANP attenuated hypoxia-induced experimental PH [56] and lowered pulmonary pressure in patients with high-altitude disease [32]. Together, these experimental and clinical studies indicate that endogenous ANP plays a physiological role in maintaining pulmonary arterial pressure homeostasis. And, furthermore, that enhancement of endogenous ANP/GC-A/cGMP signalling, for instance with drugs inhibiting ANP or cGMP degradation, may have therapeutical implications [5–7].

Pulmonary arterial remodelling in PH involves multiple vascular (EC and smooth muscle cells (SMC), adventitial fibroblasts) and nonvascular cell types (leucocytes, mast cells, platelets) [3, 21]. With the exception of platelets and leucocytes, all these cell types express the GC-A receptor [30]. Because synthetic ANP prevented acute hypoxia-induced pulmonary vasoconstriction [26, 58] and exerted direct cGMP-mediated anti-proliferative effects in cultured pulmonary arterial SMCs [24], the protective role of the ANP/GC-A/cGMP pathway in the lung circulation has mainly been attributed to its effects on pulmonary SMC. However, as shown in the present study, the GC-A receptor is also expressed at high levels in lung EC. Whereas endothelial dysfunction is central to all forms of PH [3, 21], it is unknown whether this involves impaired ANP/GC-A/cGMP signalling and how this could contribute to the progression of this disease. Therefore, the goals of this study were (1) to analyze the expression and activity of GC-A in lung endothelial cells and the impact of hypoxia; (2) to dissect the role of endothelial cells in mediating the effect of ANP in the chronic regulation of pulmonary arterial pressure by studying mice with selective disruption of the GC-A-encoding gene (*Npr1*) in endothelial cells; and (3) to elucidate the impact of endothelial ANP/GC-A dysfunction on EC inflammatory activation as well as the pulmonary levels of endothelin-1 (ET-1) and Angiotensin II (Ang II). It is known that these hormones are activated and contribute to cardiopulmonary remodelling in patients with PH [21]. On the other hand, it was shown that ANP/GC-A signalling diminishes endothelial ET-1 secretion [55] and the (inter)actions of ET-1 and Ang II in the heart and systemic circulation [19]. However, the relevance of this functional antagonism between ANP and ET-1/Ang II expression and action in the pulmonary circulation is unknown.

## Materials and methods

### Genetic mouse models

Mice with global (*GC*-*A*^−*/*−^) or endothelial cell-restricted deletion of the GC-A receptor (*GC*-*A*^*fl/fl*^; *Tie2Cre*^+*/*−^: EC GC-A KO) and their respective control littermates (*GC*-*A*^+*/*+^ or *GC*-*A*^*fl/fl*^, with unaltered GC-A expression levels) were generated and genotyped as described [33, 46]. The EC GC-A KO mice have an unaltered median life span and do not manifest clinically apparent, macroscopic changes throughout life (mice were observed until the age of 15 months). All present studies were performed with 2- to 4-month-old mice. The experiments were conducted under the guidelines on humane use and care of laboratory animals for biomedical research published by NIH (No. 85-23, revised 1996 [41]) and they were approved by the local governmental animal care committee.

### Hypoxia-induced pulmonary hypertension in mice and losartan treatment

Experimental pulmonary hypertension (PH) was induced by exposure to normobaric hypoxia. EC GC-A KO mice and littermate controls were placed into a partially ventilated plexiglass chamber (Biospherix, New York, USA), and exposed to chronic hypoxia (F_I_O_2_ 10 %, 90 % nitrogen) for 21 days under normobaric conditions [15]. Age-matched mice of both genotypes were maintained in room air and served as normoxic controls. For pharmacological blockade of the Ang II AT_1_-receptor losartan was administered via the drinking water (10 mg/kg BW/day) during 3 weeks in normoxia or hypoxia. The concentration of the drug in water was adjusted for body weight and daily water intake.

### Assessment of right ventricular pressures, pulmonary vascular remodelling and perivascular inflammation

Closed-chest right ventricular (RV) pressures were measured in anesthetized freely breathing mice (0.8–1 % isoflurane) by insertion of a 1.4 F high-fidelity pressure catheter (Millar Instruments, Houston, TX, USA) via the external jugular vein. After these invasive hemodynamic measurements, the lungs of isoflurane (1 %)-anesthetized mice were fixed with a 1 % PFA solution through the trachea at a constant pressure of 20 cmH_2_O. The trachea was ligated, and the lungs and hearts were immersed in fixative overnight. After paraffin embedding, 4 μm sections were taken along the longitudinal lung axis (ten sections per organ) and immunostained with antibodies against α-smooth muscle actin (αSMA; Sigma, Munich, Germany; dilution 1:900) and CD45 (Novus Biological, USA; dilution 1:20) to analyze the number and wall thickness of muscularized distal arteries and perivascular leucocyte infiltration [15, 47]. Vessels of 20–70 μm external diameter were classified as fully muscularized (actin staining >75 % of the circumference), partially muscularized (actin staining 25–75 % of the circumference), or nonmuscularized (<25 %). In each section, the percentage of fully or partially muscularized arteries was calculated [15]. Perivascular inflammation was assessed in the tissue section after staining for CD45 [41]. Images were captured at 40× using a Leica DM6000B microscope (Leica Instruments, Nussloch, Germany) fitted with a Leica DFC310FX digital camera. All blood vessels within lung section ranging from 20 to 70 µm were analyzed using Leica QWin software. Positively stained CD45 cells surrounding the vessels were counted [47].

### Morphometric analyses of cardiac hypertrophy

The heart was dissected to separate RV from LV plus septum (S). RV and LV + S weights were normalized to tibia lengths. Formaldehyde-fixed right (RV) and left ventricles (LV) were embedded in paraffin, and 5 µm sections were stained with hematoxylin eosin, periodic acid Schiff (PAS, to discriminate cardiomyocyte cell borders) or picrosirius red for quantification of interstitial collagen fractions. The mean cross-sectional myocyte diameters were calculated by measuring 50 (RV) to 100 (LV) longitudinally oriented myocytes with a centrally located nucleus per specimen [18, 46]. Photomicrographs were evaluated using a computer-assisted image analysis system (Olympus, Hamburg, Germany), using the analySIS software (SIS), the investigator being blinded to the genotypes [18, 46].

### Measurements of systemic arterial blood pressure and left ventricular hemodynamics

Systemic arterial blood pressure was measured by tail cuff in awake mice [33, 46]. Left ventricular (LV) function was evaluated in isoflurane-anesthetized by LV catheterization [18]. A 1.4-F combined micromanometer-tipped conductance catheter (SPR-839, Millar) was retrogradely advanced via the right carotid artery, and simultaneous recordings of LV pressure and volume were performed [18].

### Effects of ANP on cyclic GMP content of microvascular lung endothelial cells (MLEC)

Human microvascular lung (ML) EC were purchased from Promocell (Heidelberg, Germany). The cells were maintained in complete EC growth medium MV2 (Promocell) and studied at passage 4 and 5. The isolation and culture of murine MLEC has been described before [46]. Immunocytochemistry with antibodies against the endothelial marker VE-cadherin demonstrated that after the second selection, more than 95 % of cultured cells were endothelial. For the experiments the cells were seeded in gelatine-coated 6-well (for western blot) or 24-well plates (for cGMP determinations) and cultured for 48 h before synchronization in medium containing reduced serum (1 %) concentration for 24 h. The cells were thereafter exposed to 24 h hypoxia in a humidified 37 °C chamber (BioSpherix). The concentration of oxygen was reduced to 1 % by replacement with N_2_, keeping CO_2_ constant at 5 %. Control was defined as 95 % air and 5 % CO_2_. Thereafter the cells were immediately used for the extraction of membrane proteins (cell fractionation kit; Nanotools, Teningen, Germany) and for determination of cGMP responses to ANP. These steps were performed under normoxic conditions. For cGMP determinations, MLEC were pretreated with the phosphodiesterase inhibitor 3-isobutyl-1-methylxanthine (IBMX, 0.5 mmol/L, 15 min; Sigma) and then incubated with ANP (0.1 nmol/L–1 μmol/L; Bachem, Bubendorf, Switzerland) for additional 10 min. The incubation media were rapidly removed and cellular cGMP was extracted with ice-cold ethanol (70 %, v/v). After centrifugation (3000×*g*, 5 min, 4 °C), the supernatants were dried in a speed vacuum concentrator, resuspended in sodium acetate buffer (50 mmol/L, pH 6.0) and acetylated, and the cGMP content was determined by radioimmunoassay [31, 48].

### Determination of GC-A expression and activity in murine lung cell membranes

ANP-dependent guanylyl cyclase activity in crude lung cell membranes was determined as described [48]. Freshly dissected lungs were homogenized using a Polytron homogenizer in hepes buffer (HB) [25 mM HEPES (pH 7.4), 50 mM NaCl, 20 % glycerol and protease inhibitor cocktail from Roche, Mannheim, Germany]. The suspensions were pelleted by centrifugation at 45,000*g* for 20 min at 4 °C. Pellets were resuspended in HB and centrifuged two more times. To initiate cyclase activity, 40 μg membrane protein was incubated in assay buffer [25 mM/L HEPES, 4 mM/L MgCl_2_, 1 mM/L IBMX, 2 mM/L ATP, 2 mM/L GTP, 30 mM/L phosphocreatine, 400 μg/mL creatine phosphokinase (185 units/mg) and 0.5 mg/mL BSA] at 37 °C, with or without ANP. At 10 min of incubation, the reaction was stopped by addition of ice-cold ethanol (final concentration 70 % v/v). cGMP content was determined by radioimmunoassay as described above. cGMP production was normalized to protein content (40 μg/sample) and the increase in cGMP content in ANP-treated samples was compared to parallel vehicle-treated membrane preparations of the same lung.

### Western blotting

Membrane proteins from whole lungs were extracted (Thermo Scientific, Schwerte, Germany) and subjected to SDS-PAGE and immunoblotting as described [18]. The primary antibodies were against GC-A (generated in our laboratory [48]) and β-tubulin or GAPDH (for loading control; Cell Signaling, Frankfurt/Main, Germany). The blots were developed using the ECL detection system (Biozym Scientific GmbH, Hessisch-Oldendorf, Germany) and results were quantitated by densitometry (ImageQuant).

### Quantitative RT-PCR analysis of angiotensin converting enzyme (ACE), endothelin-1, intercellular cell adhesion molecule 1 (ICAM-1), vascular cell adhesion protein 1 (VCAM-1) and E-selectin mRNA expression levels

Extraction of mRNA from murine MLEC or peripheral lung tissue and reverse-transcription were performed as described using TRIzol reagent (Life Technologies GmbH, Darmstadt, Germany) and Transcriptor First Strand cDNA synthesis kit (Roche) [18]. Messenger RNA expression levels were analyzed by Real Time quantitative PCR with LightCycler Technology (LC-96; Roche) and FastStart Essential Probes Master with the following primers and probes (all from Roche): for ACE, sense: 5′-GTGGGTATCCCACTGAAACC-3′; antisense: 5′-CAGAAGGCTCCTGTGTCTGA-3′; and probe 121 (REF: 04693558001); for E Selectin, sense: 5′-TCCTCTGGAGAGTGGAGTGC-3′; antisense: 5′-GGTGGGTCAAAGCTTCACAT-3′; and probe 19 (REF: 04686926001); ET-1, sense: 5′-CTGCTGTTCGTGACTTTCCA-3′, antisense: 5′-TCTGCACTCCATTCTCAGCTC-3′, and probe 50 (REF: 04688112001); ICAM-1, sense: 5′-CGAAGCTTCTTTTGCTCTGC-3′; antisense: 5′-GTCCAGCCGAGGACCATA-3′; and probe 10 (REF: 04685091001); VCAM-1: sense: 5′-TGGTGAAATGGAATCTGAACC-3′; antisense: 5′-CCCAGATGGTGGTTTCCTT-3′; and probe 34 (REF: 04687671001). 12S ribosomal RNA served as reference gene [sense: 3′-GAAGCTGCCAAGGCCTTAGA-3′; antisense: 5′-AACTGCAACCAACCACCTTC-3′; FastStart Essential DNA Green Master (Roche)].

### Measurement of lung immunoreactive ET-1

Samples were assayed for ET-1 immunoreactivity with a specific RIA (Bachem) as described by Aguirre et al. [1]. The peptide was extracted from lung tissue by boiling in 10× (wt/vol) 1 mol/L acetic acid for 10 min. The samples were then chilled and centrifuged at 5000*g* for 10 min at 4 °C. Aliquots (0.1 mL) of supernatant were applied to Sep-PakC_18_ columns (Waters Corporation, Milford, USA). The columns were activated by 80 % acetonitrile in 0.1 % TFA followed by 0.1 % TFA. After the column was slowly washed with 10 % acetonitrile in 0.1 % TFA, samples were eluted from the column with 80 % acetonitrile in 0.1 % TFA into polypropylene tubes and evaporated to dryness in a centrifugal concentrator. The samples were reconstituted in RIA buffer and subjected to ET-1 radioimmunoassay (Bachem) according to the manufacturer’s instructions.

### Measurement of lung immunoreactive Angiotensin II

Ang II from murine lungs was extracted and measured with a commercial Ang II ELISA (Enzo Life Sciences GmbH, Lörrach, Germany) according to the manufacturer’s instructions.

### Measurement of pulmonary bradykinin-9 levels

Bradykinin was measured with an EIA Kit (Phoenix Europe, Karlsruhe, Germany). Tissue extractions and measurements were performed according to the manufacturer’s protocol. Freshly dissected lung samples were boiled in 75 % acetic acid for 20 min (1 mL/100 mg tissue), homogenized with an ULTRA-TURRAX, centrifuged (15,000*g*, 30 min, 4 °C) and the supernatants were extracted with Sep-PakC_18_ columns. The eluates were dried, reconstituted in assay buffer and subjected to Bradykinin EIA. Bradykinin levels were normalized to protein content (BCA assay).

### Statistics

Results are presented as mean ± SEM. Group comparisons were performed using either unpaired *t* test or two-way ANOVA followed by the multiple-comparison Bonferroni *t* test to assess differences between groups. *P* values of less than 0.05 were considered statistically significant. The individual sample sizes for each set of data (*n*) are provided in the figure legends.

## Results

### GC-A is expressed in lung endothelial cells and is downregulated by hypoxia

**Fig. 1:**
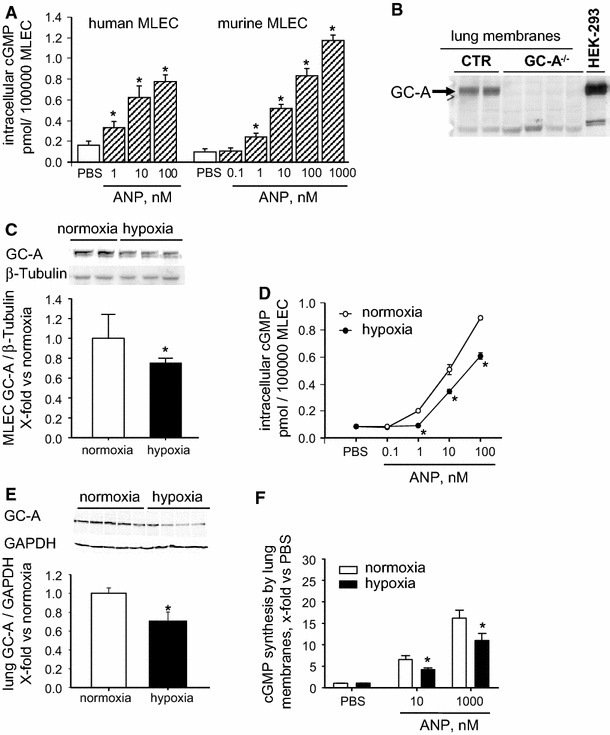
Pulmonary endothelial ANP/GC-A/cGMP signalling is attenuated by hypoxia. **a** Effect of ANP on cGMP content of cultured human (6 wells per condition; 2 independent experiments) and murine (15 dishes per condition; 5 experiments) microvascular lung endothelial cells (MLEC, 10 min incubation). **b** Representative immunoblot: strong GC-A expression (apparent MW is ~130 kDa) in cell membranes prepared from wildtype (CTR) lungs (loading 80 μg/lane). The immunoreactive signal is abolished in membranes prepared from mice with global GC-A deletion (GC-A^−/−^). Protein extracts from GC-A-expressing HEK-293 cells were used as positive control. **c**, **d** In murine MLEC, hypoxia (1 % O_2_, 24 h downregulates GC-A expression (**c**; western blots, 40 μg protein/lane) and ANP-induced intracellular cGMP synthesis (**d**) (6 wells from 3 independent experiments). **e**, **f**, In mice, chronic hypoxia (normobaric F_i_O_2_ of 10 % during 3 weeks) downregulates pulmonary membrane GC-A expression (**e**; western with 40 μg protein/lane) and ANP-stimulated lung cell membrane GC-A/cGMP activity (*n* = 6). *P < 0.05 vs. normoxia

To assess the pulmonary endothelial role of GC-A, first we tested the effects of ANP on cGMP levels of cultured human and murine microvascular lung endothelial cells (MLEC). Treatment with ANP (0.1 nmol/L–1 μmol/L, 10 min) provoked similar concentration-dependent cGMP increases in both species (Fig. 1a). Accordingly, western blot analyses revealed high lung GC-A levels in control mice (Fig. 1b). As also shown, the immunoreactive protein was not detected in lungs from mice with global GC-A deletion (GC-A^−/−^), demonstrating the specificity of our antibody [48]. Exposure of murine MLEC to hypoxia (1 % O_2_, 24 h) significantly attenuated GC-A expression (Fig. 1c) and the cGMP-responses to ANP (Fig. 1d). To study whether hypoxia-induced downregulation of lung GC-A occurs in vivo, we exposed mice to normobaric hypoxia (F_i_ 10 % O_2_) for 21 days [15]. Figure 1e, f shows that pulmonary cell membrane GC-A expression and activity were significantly impaired by chronic hypoxia.

### Endothelial cells are a main expression site of GC-A in the lung

**Fig. 2:**
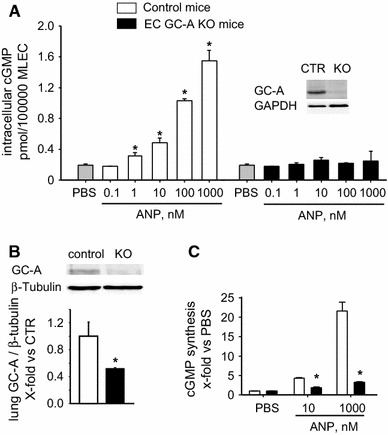
Inactivation of GC-A in lung endothelial cells of EC GC-A KO mice. **a** Effects of ANP on intracellular cGMP content of MLEC prepared from EC GC-A KO and control littermates (10 min incubation; *n* = 6 per genotype). *Inset* Representative western blot of GC-A expression in MLEC. **b** Immunoblot analyses of GC-A expression levels in whole lung protein extracts prepared from EC GC-A KO and control mice (*n* = 5). **c** Guanylyl cyclase activity assays: ANP-dependent cGMP synthesis by lung cell membranes prepared from EC GC-A KO and control mice (*n* = 6). *P < 0.05 vs. controls

To dissect the role of endothelial cells in mediating the homeostatic effect of ANP on pulmonary arterial pressure, we studied mice with conditional, endothelial-restricted disruption of GC-A (EC GC-A KO) and control littermates [46]. As shown in Fig. 2a, in cultured MLEC isolated from the KO mice GC-A expression and cGMP responses to ANP were fully abolished, demonstrating efficient endothelial GC-A deletion. Western blot analyses of whole-lung protein extracts revealed ≈60 % reduction of pulmonary GC-A protein levels in EC GC-A KO mice (Fig. 2b). Even more, ANP-stimulated GC-A activity in lung cell membranes was reduced by more than 80 % (Fig. 2c). As already mentioned, different cell types in the lung express the GC-A receptor. Considering that EC make up ~30 % of the lung cells [13], our studies of control and EC GC-A KO mice indicate that endothelia are one main expression site of GC-A in the lung.

### Genetic deletion of endothelial GC-A in mice causes PH and pulmonary vascular remodelling

**Fig. 3:**
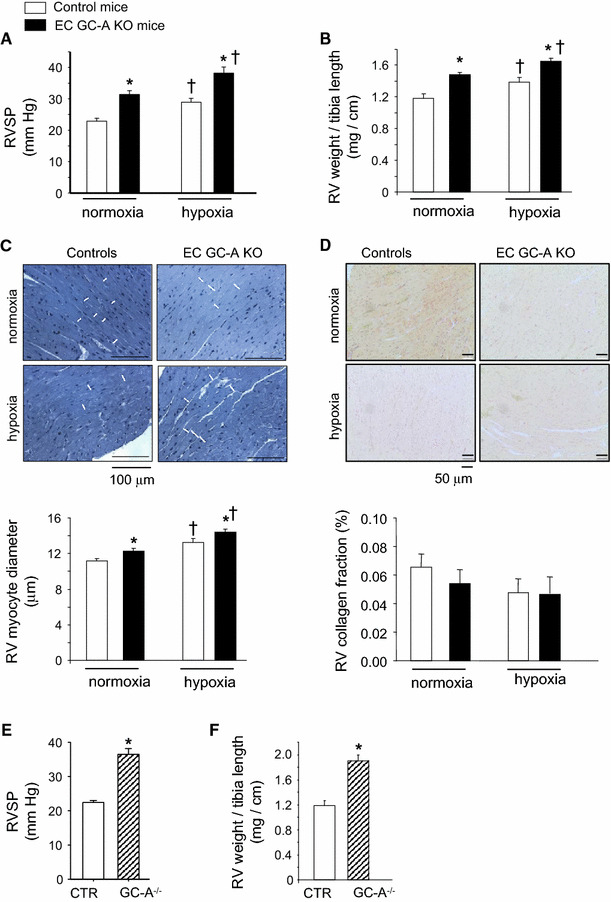
Genetic deletion of the endothelial GC-A receptor in mice causes pulmonary hypertension and right ventricular (RV) hypertrophy under normoxic conditions and, more, after chronic hypoxia (F_i_O_2_ 10 % during 3 weeks). **a** Elevated RV systolic pressures (SP) in EC GC-A KO mice compared to respective controls under normoxia and after hypoxia. **b**, **c** Ratios of RV weight to tibia length and RV myocyte diameters (indicated by *white lines* in longitudinal PAS stained sections) were increased in EC GC-A KO mice. Hypoxia further enhanced RV hypertrophy of EC GC-A KO mice. **d** Picrosirius *red* stainings revealed that RV interstitial collagen fractions were not different between genotypes and conditions (*n* = 8 mice per group); **e**, **f** Increased RVSP and enhanced RVW/tibia length ratios in mice with global, systemic GC-A deletion (GC-A^−/−^) compared to respective controls (CTR) (*n* = 6 mice per genotype studied under normoxia). *P < 0.01 vs. controls; ^†^ *P* < 0.01 vs. normoxia

To study the impact of endothelial GC-A dysfunction on pulmonary arterial pressure we compared RV pressures in anesthetized EC GC-A KO and control littermates. RV catheterization revealed that EC GC-A KO mice have increased RV systolic pressures (RVSP; Fig. 3a). This was accompanied by RV hypertrophy, with enhanced RV weight/tibia length ratios (Fig. 3b) and greater RV myocyte diameters (Fig. 3c depicts the mean cross-sectional diameters of RV myocytes with a centrally located nucleus). Picrosirius red stainings did not reveal signs of RV interstitial fibrosis (Fig. 3d). Together these observations indicate that EC GC-A KO mice have mild but consistent PH already under normoxic conditions. This phenotype was independent of age (2- to 8-month-old mice were studied) and gender. Notably, peak RVSP values in EC GC-A KO mice nearly reached the levels of mice with global, systemic GC-A deletion (GC-A^−/−^ mice [33], see Fig. 3e), whereas RV hypertrophy was more pronounced in the later genotype (Fig. 3f). Exposure to chronic hypoxia induced PH and RV hypertrophy in EC GC-A KO and control littermates, again with greater RVSP and RV hypertrophy in the former, without significant RV fibrosis (Fig. 3a–d). However, the absolute increase in mean RVSP in response to hypoxia was not greater in EC GC-A KO mice than that in controls (+7.5 vs. 6.6 mmHg, respectively). Hypoxia-induced hematocrite raises did not differ between genotypes (controls 0.47 ± 0.01 % (normoxia) vs. 0.6 ± 0.01 %* (hypoxia); EC GC-A KO 0.46 ± 0.01 vs. 0.57 ± 0.08 %*; *P < 0.05 vs. normoxia).

**Fig. 4:**
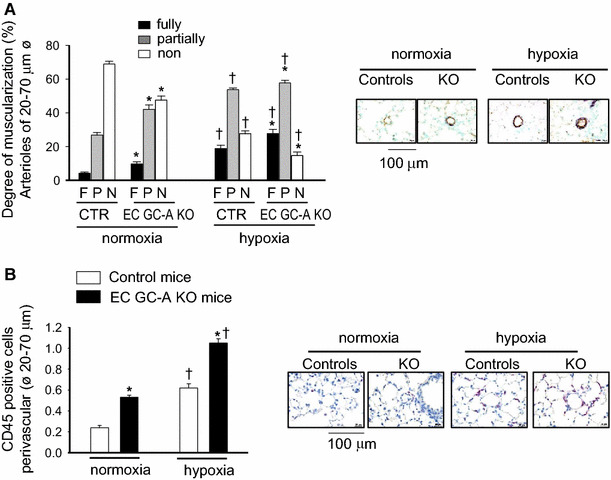
Genetic deletion of the endothelial GC-A receptor in mice causes pulmonary vascular remodelling together with mild perivascular inflammation under normoxic conditions and, more, after chronic hypoxia. Lung sections were immunostained for SMC α-actin or for lymphocyte common antigen (CD45). **a** Quantification of the relative numbers of fully (F), partially (P) and non (N) muscularized arterioles and **b** of perivascular CD45-positive cells per field demonstrated enhanced pulmonary vascular remodelling and perivascular inflammatory infiltration in EC GC-A KO mice under normoxia and after hypoxia (*n* = 8 mice per group). *P < 0.01 vs. controls; ^†^ *P* < 0.01 vs. normoxia

To investigate the effect of endothelial GC-A ablation on pulmonary vascular remodelling, the degree of muscularization of peripheral arterioles was analyzed by immunostainings with anti-α-SMA antibodies [15]. Morphometrical analyses showed an increase in the relative number of fully and partially muscularized vessels and a concomitant decrease of nonmuscularized vessels in EC GC-A KO as compared with control lungs (Fig. 4a). In addition, immunostainings with anti-CD45 antibodies [47] revealed mild perivascular leucocyte infiltration (Fig. 4b). Hypoxia provoked lung vascular remodelling and perivascular inflammation in control and, significantly more, in EC GC-A KO mice (Fig. 4a, b). Again, the relative changes (as compared to normoxia) were similar in both genotypes.

### Pulmonary hypertension in EC GC-A KO mice is not secondary to left heart disease

**Fig. 5:**
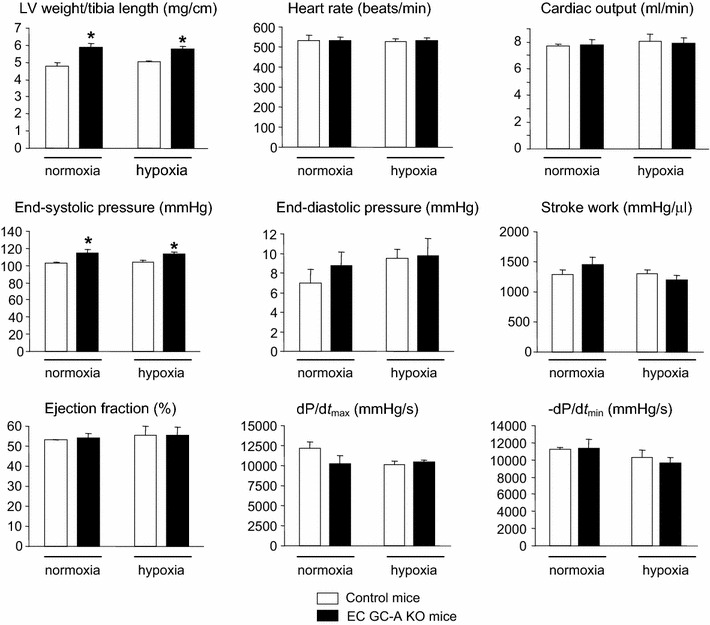
Left ventricular (LV) weight and function of anesthetized control and EC GC-A KO mice determined by pressure–volume analyses after normoxia or chronic hypoxia. Ratios of LV weight to tibia length and LV systolic pressures were similarly increased in EC GC-A KO mice under normoxic and after hypoxic conditions. All other parameters of LV contraction and relaxation were not different between genotypes and conditions. *n* = 6 mice per genotype and condition; *P < 0.05 vs. controls

In agreement with our previous report [40], EC GC-A KO mice used in the present study had mild systemic hypertension and subtle LV hypertrophy without fibrosis (Table 1). The degree of LV hypertrophy was not changed after hypoxia (Fig. 5). Pressure–volume relationships (studied by LV catheterization) demonstrated that LV contractile and relaxation functions of EC GC-A KO mice were unaltered (Fig. 5). LV end-systolic pressures were slightly greater, consistent with the mildly enhanced afterload. As also shown in Fig. 5, hypoxia did not influence LV function in control or EC GC-A KO mice. In addition the lung wet-to-dry weight ratios were equal for EC GC-A KO and controls (Table 1). Together, these data indicate that the PH of EC GC-A KO mice is not secondary to LV dysfunction.

**Table 1:** EC GC-A KO mice have subtle systemic arterial hypertension and mild left ventricular (LV) hypertrophy without fibrosis

	Controls	EC GC-A KO
SBP (mmHg)	118 ± 2	133 ± 3*
DBP (mmHg)	75 ± 3	82 ± 3*
HR (bpm)	589 ± 18	564 ± 13
Body weight (g)	25 ± 1.5	25 ± 1.2
Heart weight (mg)	118 ± 4.5	145 ± 6*
LV weight/tibia length (mg/cm)	4.78 ± 0.19	5.9 ± 0.2*
LV myocyte diameter (μm)	12 ± 0.4	14.8 ± 0.8*
Collagen fraction (%)	0.1 ± 0.02	0.11 ± 0.03
Lung wet/dry weight	4.4 ± 0.04	4.5 ± 0.04
Hematocrite (%)	43 ± 1.7	42 ± 2.4

Systemic systolic (SBP) and diastolic (DBP) arterial blood pressure, heart rate (HR) (determined by tail cuff), hematocrite and LV morphology (necropsy and histology) of EC GC-A KO and control mice. *n* = 8, * *P* < 0.05 vs. control littermates

### Pulmonary levels of immunoreactive endothelin-1 are not altered in EC GC-A KO mice

**Fig. 6:**
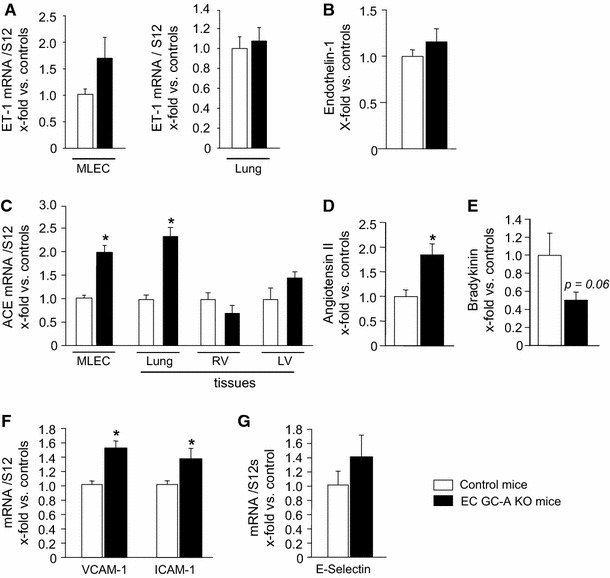
Unaltered endothelin-1 but altered levels of angiotensin converting enzyme (ACE), Ang II, bradykinin and EC adhesion molecules in cultured microvascular lung endothelial cells (MLEC) and/or in lungs of EC GC-A KO mice. **a**, **b** Real-time RT-PCR and radioimmunoassay (RIA) showed that the endothelial and pulmonary mRNA and peptide levels of ET-1 were not significantly different between genotypes. **c** ACE mRNA expression was increased in GC-A-deficient MLEC and in lungs from EC GC-A KO mice (*n* = 5). **d**, **e** Pulmonary levels of immunoreactive Ang II were significantly greater in EC GC-A KO mice whereas the pulmonary levels of bradykinin were diminished (*P* = 0.08). **f** VCAM-1, ICAM-1 and E-Selectin mRNA levels were increased in lungs from EC GC-A KO mice. The mRNA levels of all target genes were normalized to the levels of 12S ribosomal RNA as reference gene. All data are illustrated as *x*-fold changes in EC GC-A KO vs. control mice. *n* = 8 per genotype; *P < 0.05 vs. controls

To elucidate the mechanism(s) contributing to PH in EC GC-A KO mice we studied specific ANP-modulated pathways known to be altered in clinical PH. In particular, ET-1 levels are upregulated in patients with PH and endothelin receptor antagonists are used in its treatment [45]. Synthetic ANP inhibits ET-1 release from cultured human umbilical venous endothelial cells [55]. Therefrom, we hypothesized that endothelial GC-A dysfunction leads to increased lung ET-1 levels which, via the vasoconstrictory and SMC proliferative actions of this peptide, could contribute to PH in EC GC-A KO mice. However, qRT-PCR did not reveal significant differences of the ET-1 mRNA levels in GC-A-deficient MLEC and in lungs from EC GC-A KO mice in comparison to controls (Fig. 6a). Even more, pulmonary ET-1 levels did not differ between genotypes (Fig. 6b).

### Enhanced expression levels of ACE and of endothelial adhesion molecules in EC GC-A KO lungs

Experimental and clinical studies indicate that the renin–angiotensin–aldosterone system (RAAS) is involved in the pathophysiology of PAH [12, 14, 35, 37, 38, 43]. Synthetic, exogenous ANP attenuates the expression of angiotensin converting enzyme (ACE) and counterregulates the cardiovascular effects of Ang II [17, 19, 27, 52]. Thus, we evaluated whether the ACE/Ang II pathway participates in PH of EC GC-A KO mice. Indeed, qRT-PCR revealed increased ACE expression in GC-A-deficient MLEC and lungs from EC GC-A KO mice (Fig. 6c). As also shown, ACE mRNA expression was unaltered in other tissues from the KO mice such as heart. The direct effect of the dipeptidyl peptidase ACE is to increase levels of Ang II and decrease levels of bradykinin. To follow the hypothesis that increased Ang II together with decreased local bradykinin levels contribute to PH of EC GC-A KO mice we determined the lung levels of these peptides. Indeed, levels of immunoreactive Ang II were greater in EC GC-A KO lungs (Fig. 6d). Concomitantly, the levels of bradykinin-9 were attenuated although, due to high variability, the difference to control lungs did not reach statistical significance (*P* = 0.08; Fig. 6e). Lastly, qRT-PCR revealed increased pulmonary expression of the EC adhesion molecules VCAM-1 and ICAM-1 and mild not-significant increases of E-selectin in EC GC-A KO mice (Fig. 6f, g).

### Enhanced ACE/Angiotensin II signalling contributes to PH of EC GC-A KO mice

**Fig. 7:**
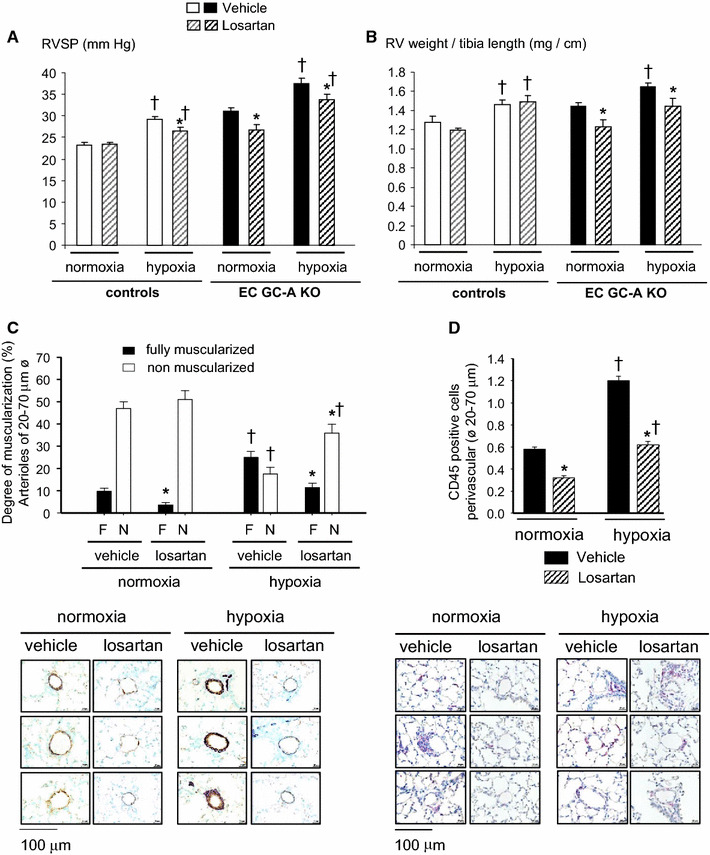
Blockade of the Ang II/AT_1_-receptor reversed the pulmonary vascular changes in EC GC-A KO mice. **a** Treatment of control mice with losartan (10 mg/kg BW/day during 3 weeks) had no effect on baseline RVSP (normoxia) but attenuated the increment by chronic hypoxia. In EC GC-A KO littermates losartan decreased elevated RVSP under normoxic conditions and attenuated the increment by chronic hypoxia. **b** In control mice losartan did not prevent hypoxia-induced RV enlargement. However, losartan reversed baseline RV hypertrophy (under normoxia) in EC GC-A KO littermates and prevented the increase by hypoxia. **c** The number of fully muscularized lung arterioles, and **d** surrounding infiltration by CD45-positive leucocytes in EC GC-A KO mice under normoxia and after hypoxia were significantly decreased by losartan. *n* = 6–9 mice per group; *P < 0.05 vs. vehicle; ^†^ *P* < 0.05 vs. normoxia

To study whether increased lung ACE/Ang II levels contribute to the PH of EC GC-A KO mice, we compared the effects of chronic blockade of the Ang II/AT_1_-receptor in both genotypes. Figure 7 illustrates the impact of losartan treatment (10 mg/kg/day, 3 weeks) on RVSP (Fig. 7a) and on the ratios of RV weight/tibia length (Fig. 7b) of mice maintained under normoxia or hypoxia. As illustrated, losartan did not affect these parameters in normoxic controls. The drug partly prevented the increases in RVSP of control mice subjected to hypoxia (Fig. 7a); however, this did not ameliorate either the hypertrophy of the RV (Fig. 7b) or the thickening of the distal pulmonary arteries. The percentage (%) of fully muscularized distal arteries was: 0.96 ± 0.44 in control mice under normoxia; 6.84 ± 1.6* in controls after hypoxia; and 5.1 ± 1.2* in controls treated with losartan during hypoxia (*n* = 6 mice per group; *P < 0.05 vs. normoxia).

Notably, while losartan had no appreciable effects in normoxic control mice, it almost reversed the baseline pulmonary hypertension of EC GC-A KO mice. This is indicated by the decreases of RVSP (Fig. 7a), RV hypertrophy (Fig. 7b), pulmonary vascular remodelling and perivascular inflammation (Fig. 7c, d). In addition, administration of losartan during hypoxia partly but significantly prevented the hypoxia-driven augmentation of these cardiovascular changes (Fig. 7a–d). Lastly losartan also reversed the mild (hypoxia-independent) LV hypertrophy of EC GC-A KO mice, as indicated by the following LV weight/tibia length ratios (in mg/cm): 5.9 ± 0.2 (KO, normoxia, vehicle); 3.9 ± 0.11* (KO, normoxia, losartan); 5.8 ± 0.14 (KO, hypoxia, vehicle); 3.8 ± 0.27* (KO, hypoxia, losartan) (*n* = 9 mice per group; *P < 0.05 vs. vehicle). Together these observations indicate that AT_1_ receptor signalling has a significant role in the cardiac and pulmonary remodelling changes of EC GC-A KO mice.

## Discussion

Together with previous reports [32, 56–58], our experimental studies demonstrate that ANP, via its GC-A receptor, plays an important physiological role in the moderation of pulmonary arterial pressure and lung vascular remodelling under normoxic and hypoxic conditions. The major novel findings are (1) EC are a major expression site of the GC-A receptor in the lung; (2) hypoxia impairs pulmonary endothelial GC-A expression and signaling; (3) genetic inactivation of the endothelial GC-A receptor in mice (EC GC-A KO) provokes PH, pulmonary vascular remodeling and subtle perivascular inflammatory infiltration already under normoxic conditions; (4) peak RVSP values in EC GC-A KO mice were similar to the levels of mice with deletion of GC-A in all cell types (GC-A^−/−^), indicating that the endothelial effects of ANP are critically involved in the chronic moderation of pulmonary arterial pressure and vascular homeostasis by this hormone, at least in the murine system; and (5) enhanced local ACE/Ang II signaling contributes to the pulmonary vascular alterations in mice with endothelial GC-A dysfunction.

The increases in RVSP and the extent of pulmonary vascular remodeling in mice with global ANP or GC-A inactivation [28, 29], or endothelial-restricted GC-A ablation are very consistent. In fact, less pronounced and more variable changes were observed in other disease-relevant genetic mouse models. For instance, wide ranges of RVSP were observed in mice with endothelial deletion of the BMPR2 gene (20.7–56.3 mmHg; median, 27 mmHg) compared with control mice (19.9–26.7 mmHg; median 23 mmHg), and only a subset of BMPR2-deficient mice with RVSP >30 mmHg exhibited RV hypertrophy and pulmonary vascular remodeling [23]. Even more, exposure of wild type rats or mice to chronic hypoxia (as accepted experimental model of PH) increases RVSP by 7–10 mmHg [15, 28, 29]. Hence, in general the functional and morphological pulmonary alterations in experimental PH are much less pronounced as in the clinical setting, emphasizing that patients have a multifactorial disease whereas experimental studies attempt to dissect the contribution of specific genes or mechanisms. The present experimental study suggests that endothelial ANP/GC-A dysfunction could be one aspect of the complex neurohumoral imbalance accompanying and aggravating PH, in particular hypoxia-induced PH in chronic high-altitude disease. Our observations may stimulate clinical studies to follow this possibility.

Experimental and clinical studies showed that during chronic hypoxia, right heart ANP and BNP synthesis and circulating NP levels increase, possibly in response to the RV pressure overload provoked by pulmonary vasoconstriction [9–11, 44]. Because synthetic ANP counterregulates hypoxic pulmonary vasoconstriction [8, 22, 26] and limits the interaction of endothelial and inflammatory cells [25, 39] and the proliferation of cultured vascular SMC [24], it was proposed that enhanced endogenous ANP/BNP release helps to mitigate the development of hypoxic PH [9, 10]. However, as shown here, hypoxia can decrease lung GC-A levels and endothelial GC-A/cGMP responses to ANP, which will attenuate these protective ANP (and BNP) effects. The inhibition of ANP/GC-A signaling by hypoxia has also been observed in coronary EC [2] but the molecular mechanism is presently unknown and requires further study.

Endothelial GC-A dysfunction might cause PH in mice by provoking chronic increases in pulmonary arteriolar tone and/or vascular remodelling. Hence, we hypothesized that ANP physiologically regulates the endothelial release or (in) activation of factors locally modulating these processes, such as ET-1, Ang II or bradykinin. And, conversely, that this effect of ANP is abolished in EC GC-A KO mice. Interestingly, whereas ET-1 mRNA and protein levels were unaltered, ACE mRNA levels were increased in GC-A-deficient MLEC and in lungs from EC GC-A KO mice. Concomitantly, pulmonary Ang II levels were greater in the mutants whereas bradykinin levels tended to be diminished. It is well known that Ang II, via AT_1_ signalling, not only causes vasoconstriction, but also migration and proliferation of SMC as well as recruitment of inflammatory cells [16, 54]. Specifically, inhibition of ACE decreased the cellular inflammatory response in experimental models of lung inflammation [4]. Indeed, in the present study AT_1_-receptor blockade with losartan largely reversed PH, pulmonary vascular remodelling and inflammation in normoxic EC GC-A KO mice. Even more, losartan significantly attenuated the exacerbation of these cardiovascular changes in response to hypoxia. Together these observations indicate that enhanced ACE-dependent local Ang II formation contributes to these phenotypical alterations. In line with our results, several experimental and clinical studies have implicated the involvement of the RAAS in the pathogenesis of PH [35]. All components, including renin, angiotensinogen, ACE and both subtypes of Ang II receptors, are expressed in the lung [38, 43]. Increased ACE expression and activity in the endothelium of peripheral pulmonary arteries have been found in animal models of PH and, importantly, in patients with various forms of PAH [38, 43]. However, the pathophysiological mechanism(s) remain(s) unclear. Our studies add a novel piece of information showing that pulmonary endothelial ANP/GC-A/cGMP-dysfunction is associated with enhanced ACE expression and activity. The inhibition of ACE levels by ANP was also observed by others [52] and we will try to clarify the mechanism in our future investigations.

Notably, in the present study losartan did not clearly ameliorate hypoxic pulmonary hypertension in control mice. The increase in RVSP was only partly prevented, whereas RV hypertrophy and lung vascular remodelling were not at all attenuated by the drug. Hence, mechanisms independent of the AT_1_ receptor seem to predominate. In line with our observations, blockade of the AT_1_ receptor by olmesartan [49] or genetic deletion of ACE [53] also failed to ameliorate hypoxic PH and RV hypertrophy in mice. In contrast, AT_1_ antagonists (GR138950C, olmesartan) reversed hypoxia-induced cardiopulmonary remodelling in rats [40, 57]. The discrepancy between these results remains unexplained; species differences might be involved.

Beside increased Ang II diminished bradykinin levels may contribute to PH and lung perivascular inflammation of EC GC-A KO mice. The small nine amino-acid vasoactive peptide bradykinin has dual roles by exerting pathophysiological as well as beneficial physiological effects, mainly by stimulation of bradykinin B2 receptors. Specifically in the lung, inhibition of bradykinin metabolic breakdown by ACE inhibitors or exogenous administration of B2 receptor agonists exerted protective effects, reducing pulmonary arterial pressure in experimental hypertension [50] and neutrophil recruitment by lipopolysaccharide [4]. These protective effects of bradykinin involve the endothelial release of NO, prostacyclin and tissue-type plasminogen activator [4]. Hence, we hypothesize that PH and perivascular inflammation in EC GC-A KO mice is mediated through both a local increase in Ang II and a decrease in bradykinin mediated signalling.

In general, experimental and clinical studies emphasize that a compromised endothelial barrier plays a central role in the pathogenesis of PH [3, 45]. In fact, both acute and chronic hypoxia in mice and rats induce subtle but significant inflammation in the lung *prior* to the onset of structural changes in the vessel wall [34, 36]. On the other hand, numerous studies in vitro/in vivo indicated that ANP exerts pulmonary endothelial barrier-protecting actions. Synthetic ANP reduced hypoxia, TNF-α, thrombin, or bacterial endotoxin (PepG)-induced paracellular hyperpermeability of pulmonary microvascular and macrovascular endothelial cells cultured on permeable supports and acute PepG-induced lung injury in mice [30, 51]. Conversely, enhanced PepG-induced lung injury, ICAM-1/VCAM-1 expression and vascular leak were observed in ANP^−/−^ mice [51]. We did not observe macroscopic signs of pulmonary edema in EC GC-A KO mice under normoxic or hypoxic conditions. However, we found increased pulmonary levels of the endothelial adhesion molecules ICAM-1 and VCAM-1. Together with the imbalance between Ang II and bradykinin signalling these changes possibly contribute to enhanced pulmonary neutrophil infiltration and PH in EC GC-A KO mice.

### Study limitations

One limitation of the EC GC-A KO mice is that the GC-A receptor is absent not only in pulmonary but also in systemic endothelia. Unfortunately, a selective disruption of target genes within the pulmonary circulation is technically impossible so far and therefore this limitation is shared by other disease relevant genetic mouse models [20]. Hence, because EC GC-A KO mice have mild systemic arterial hypertension and subtle LV hypertrophy, it is possible that PH was secondary to the systemic phenotype. However, invasive haemodynamic studies clearly demonstrated that cardiac output and LV function of EC GC-A KO mice are unaltered, also after chronic hypoxia. In addition there are no signs of pulmonary edema, corroborating that the PH of these mice is not secondary to left ventricular failure. Even more, we did not observe vascular thickening or inflammation in other tissues of EC GC-A KO mice. Together these observations indicate that the pulmonary vascular alterations of EC GC-A KO mice are not secondary to systemic changes.

Concordant to the mice with systemic ANP or GC-A deletion ([28, 29] and present study), mice with EC-restricted GC-A ablation have mild PH already under baseline, normoxic conditions, which was aggravated by chronic hypoxia. However, the absolute increases in mean RVSP and in vascular remodelling in response to CH were similar in EC GC-A KO mice and in controls. Again this is consistent with previous observations in mice with global ANP or GC-A inactivation [28, 29]. Hence, it remains impossible to definitively determine whether ANP/GC-A dysfunction aggravates hypoxic PH or merely produces normoxic PH that is then amplified by hypoxia.

## Conclusion

In summary, endothelial effects of ANP play a critical physiological role in the chronic maintenance of pulmonary vascular homeostasis. Our observations in vitro and in EC GC-A KO mice suggest that ANP moderates the endothelial expression (VCAM-1, ICAM-1) or formation of local factors (ACE/Ang II, possibly bradykinin) regulating SMC proliferation and the interaction of EC and inflammatory cells. Our experimental observations in a monogenetic mouse model suggest that chronic endothelial ANP/GC-A dysfunction, e.g. provoked by hypoxia, might contribute to lung endothelial barrier impairment and vascular remodelling, and thereby to PH.
