# Correction to: Endothelial actions of atrial natriuretic peptide prevent pulmonary hypertension in mice

**DOI:** 10.1007/s00395-022-00916-9

**Published:** 2022-02-28

**Authors:** Franziska Werner, Baktybek Kojonazarov, Birgit Gaßner, Marco Abeßer, Kai Schuh, Katharina Völker, Hideo A. Baba, Bhola K. Dahal, Ralph T. Schermuly, Michaela Kuhn

**Affiliations:** 1 Physiologisches Institut der Universität Würzburg, Röntgenring 9, 97070 Würzburg, Germany; 2 Department of Internal Medicine, University of Gießen and Marburg Lung Center (UGMLC), Justus-Liebig University Gießen, Giessen, Germany; 3 German Center for Lung Research, Heidelberg, Germany; 4 Institute of Pathology, University Hospital of Essen, University of Duisburg-Essen, Essen, Germany

## Correction to: Basic Res Cardiol (2016) 111:22 10.1007/s00395-016-0541-x

When this article was initially published, two of the illustrative pictures in Fig. 7 had been duplicated from Fig. 4. This mistake does not affect the results and conclusions. The figure should have appeared as shown below.
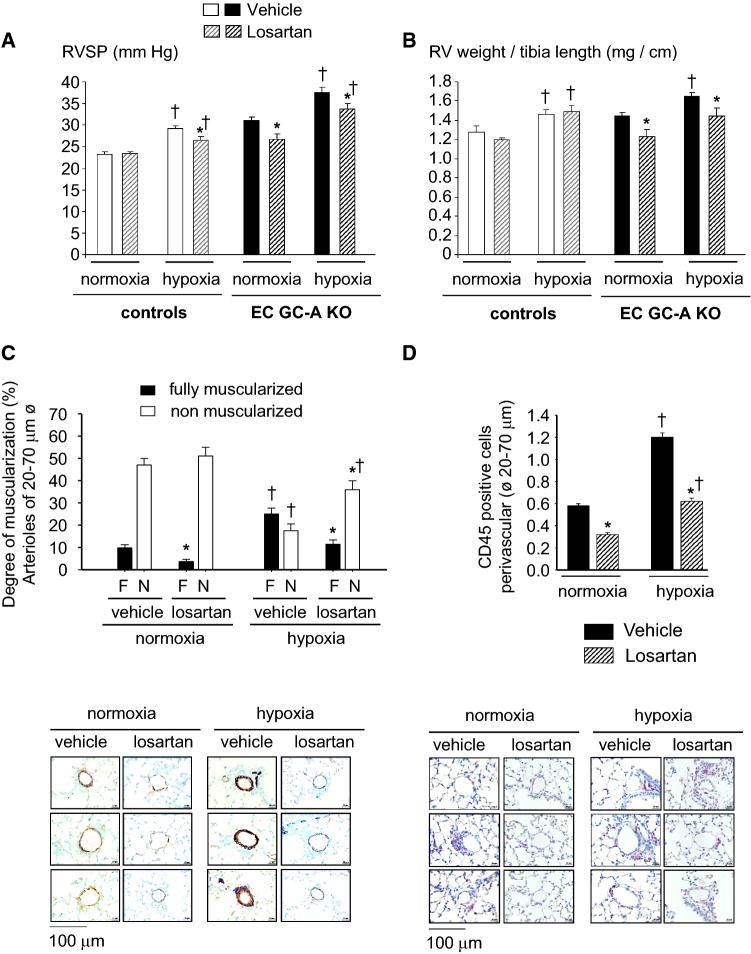


The original article has been corrected.

